# Outer-synchronization criterions for asymmetric recurrent time-varying neural networks described by differential-algebraic system *via* data-sampling principles

**DOI:** 10.3389/fncom.2022.1029235

**Published:** 2022-11-17

**Authors:** Ping Li, Qing Liu, Zhibing Liu

**Affiliations:** ^1^College of Commercial, Pingxiang University, Pingxiang, China; ^2^College of Mathematics and Statistics, Huanggang Normal University, Huanggang, China

**Keywords:** asymmetric recurrent time-varying neural networks, differential-algebraic system, singular neural networks, data-sampling, outer-synchronization

## Abstract

Asymmetric recurrent time-varying neural networks (ARTNNs) can enable realistic brain-like models to help scholars explore the mechanisms of the human brain and thus realize the applications of artificial intelligence, whose dynamical behaviors such as synchronization has attracted extensive research interest due to its superior applicability and flexibility. In this paper, we examined the outer-synchronization of ARTNNs, which are described by the differential-algebraic system (DAS). By designing appropriate centralized and decentralized data-sampling approaches which fully account for information gathering at the times *t*_*k*_ and $t_{k}^{i}$. Using the characteristics of integral inequalities and the theory of differential equations, several novel suitable outer-synchronization conditions were established. Those conditions facilitate the analysis and applications of dynamical behaviors of ARTNNs. The superiority of the theoretical results was then demonstrated by using a numerical example.

## 1. Introduction

A novel approach to artificial general intelligence (Yang et al., 2021a,b, 2022a,b,c) are critical studies in the field of brain-inspired intelligence to realize high-level intelligence, high accuracy, high robustness, and low power consumption in comparison with state-of-the-art artificial intelligence works. The research of neural networks can promote or accelerate the development of artificial intelligence. Due to their dynamic complexity and a vast range of civil and military applications, neural networks (NNs) garner a great deal of interest, such as associative memory, classification, identification, and optimized calculations (Hu and Hu, 2019; Zhang et al., 2021a; Lv et al., 2022). With the widespread application of NNs and the expansion of study, numerous varieties of NNs have been proposed by researchers. Consider conventional NNs (CNNs), feedforward NNs, and recurrent NNs (RNNs), for instance. RNNs are mostly employed to simulate machine learning (Cho et al., 2014; Shi et al., 2017) and language processing (Mao et al., 2014; Yin et al., 2017). Combining differential equations with RNNs yields asymmetric RNNs (ARNNs) (Chang et al., 2019). Currently, relatively few studies have been conducted on ARNNs. Ansari (2022) suggested an ARNN with a single layer for solving linear equations. This research also provides a straightforward method for setting various connection weights. Lu et al. (2016) explored outer-synchronization of ARNNs *via* a data-sampling control mechanism. For more related works on the network, refer to Liu et al. (2019, 2020, 2021), Lv et al. (2022a,b), and Zhang et al. (2020, 2021b).

The differential-algebraic system (DASs) are a broader scope of modeling systems, sometimes known as singular systems or restricted systems. DASs consist of differential and algebraic equations (AEs), the latter of which illustrates the limitations of systems. General DASs can be used to model power systems, while stochastic DASs are used to simulate just minor changes in transmission line parameters and system loads (Federico and Zárate-Miñano, 2013). This category includes aircraft flight trajectory tracking, optimization control systems, and crafting processes. DASs have superior modeling and simulation resulting in physics and engineering compared to differential dynamical systems. Consequently, research on the applications and theory of DASs is proliferating. A study of the Lyapunov stability of equilibria in DASs is presented (Bill and Mareels, 1990). Constantinos (1988) determined whether a requirement for additional restrictions satisfied by initial values can be derived from the differentiation of a subset of nonlinear DAS equations. In Esposito and Floudas (2000), two global optimization techniques for DAS parameter estimation were suggested. Non-regular linear DAS state estimation and dynamic feedback stabilization have been investigated (Berger and Reis, 2017).

Synchronization is the dynamic behavior of complex system interactions. It refers to changes in the rhythm of a self-sustained periodic oscillator as a result of weak interactions. There are various types of synchronization, such as quasi, complete, identical, finite-time, and generalized. There is no loss of commonality among the various definitions of quai-synchronization, which is when a system can start from any beginning value and eventually converge to an error bound with t. Any two system solutions that are in identical synchronization will converge to zero with time t. The system reaches finite-time synchronization reflecting the limit of system error is equal to zero in a finite time. Due to their varying control methodologies, various systems exhibit various synchronization styles. Synchronization, in general, is a steady state of equilibrium inside a system or between the master-slave system. For NNs, multi-agents, and DASs, synchronization as a key research hot topic yielded numerous useful outcomes. Using the features of Mittag-Leffler functions and stochastic matrices, Liu et al. (2018) derived two sets of necessary requirements for the global synchronization of a connected fractional-order system. Wu et al. (2019) suggested a discrete-time-based periodic intermittent observation control to investigate the synchronization of stochastic NNs. The fix-time synchronization problem of discontinuous fuzzy inertial NNs under uncertainty parameters is studied by constructing a new type of discontinuous control input and applying the Lyapunov-Krasovskii functional technique (Kong et al., 2021). Chen et al. (2021) coupled aperiodic intermittent control with event-triggered control, investigated the quasi-synchronization problem of CDMNN, and derived an effective criterion.

Sampled-data control is discrete rather than continuous-time when delivered across a network (Chen and Han, 2013). By building a proper control mechanism, sampling data control utilizing only partial data reduces communication costs significantly. The primary sampling control techniques for the corresponding system primarily rely on the discrete-time method, the impulsive system method, and the input delay method. The selection of the sampling interval is a crucial component of sampling control. How to use discrete sampling data control to accomplish the goals of the control system is the key problem of the research on the condition that the sampling interval is as large as possible. In recent years, the control problem of sampled data system has attracted interest of scholars. The event-triggered strategy control based on sampled data, for example, has the consensus of researchers (Su et al., 2017). The sampling interval setting is crucial to the control mechanism (Syed Ali et al., 2019). How to build the most efficient and cost-effective sampling-based control system is thus a topic worthy of investigation. For example, Liu et al. (2017) offered a stored sampled data control strategy with constant signal propagation delay to solve the stabilization problem of T-S fuzzy systems by developing a Lyapunov functional.

Based on the above analysis, this research uses centralized and decentralized data-sampling principles to explore the conditions for the ARTNN to achieve outer-synchronization. Utilizing efficient procedures to maximize access to information with limited resources is an improvement. To develop a reasonable solution, we thoroughly evaluated the system's structural characteristics and state variables in accordance with the centralization and decentralization principles and selected the optimal sampling interval. The practical conditions for the outer-synchronization of the system are determined from the characteristics of the DAS. The methodology in this paper has promoted DAS research.

The remaining sections of the work are structured as stated below. Section 2 offers the introduction and problem statement. Section 3 lists principal results and evidence. Section 4 illustrates the simulations. Section 5 concludes the article.

## 2. Preliminaries

In this section, a DANN model is established. Some basic definitions and one useful lemma are presented.

Enlightened by the frame work of Esposito and Floudas (2000) and Berger and Reis (2017), the singular ARTNN is expressed as follows:


(1) $$\begin{array}{l} E\frac{dx_{i}\left(t\right)}{dt}=-Cx_{i}\left(t\right)+A\overset{n}{\underset{j=1}{\sum }}f_{j}\left(x_{j}\left(t\right)\right)+J_{i}\left(t\right), \end{array}$$

where $x\left(t\right)=\left(x_{1}\left(t\right),\cdots ,x_{n}\left(t\right)\right)\in R^{n}$ is the neuron state vector. E is a singular constant matrix, and we suppose that 0 < *rank*(*E*) = *r* < *n*. *C* is the state coefficient matrix with respect to time t and *A* is the connection weight matrix between neurons. *C, A* ∈ *R*^*n*×*n*^ are regular. *J*_*i*_(*t*) is the external input.

Model (1) is a singular NN with implicit constraints, how to express constraints explicitly and explore the properties of such systems is a challenging task.

We assume that $C=\left(\begin{array}{cc} c_{r}\left(t\right) & 0\\ 0 & c_{n-r}\left(t\right) \end{array}\right)$, $A=\left(\begin{array}{cc} a_{r,s}\left(t\right) & a_{r,n-s}\left(t\right)\\ a_{n-r,s}\left(t\right) & a_{n-r,n-s}\left(t\right) \end{array}\right)$ and $E=\left(\begin{array}{cc} I_{r} & 0\\ 0 & 0 \end{array}\right)$. *I*_*r*_ is a *r* × *r* identity matrix.

Then, the model (1) is equivalent to the following system


(2) $$\begin{array}{l} I_{r}\frac{dx_{i}^{\left(1\right)}\left(t\right)}{dt}=-c_{r}\left(t\right)x_{i}^{\left(1\right)}\left(t\right)+a_{r,s}\left(t\right)f_{j}^{\left(1\right)}\left(x_{j}^{\left(1\right)}\left(t\right)\right)\\ \;+a_{r,n-s}\left(t\right)f_{j}^{\left(2\right)}\left(x_{j}^{\left(2\right)}\left(t\right)\right)+J_{i}^{\left(1\right)}\left(t\right), \end{array}$$

(3) $$\begin{array}{l} 0=-c_{n-r}\left(t\right)x_{i}^{\left(2\right)}\left(t\right)+a_{n-r,s}\left(t\right)f_{j}^{\left(1\right)}\left(x_{j}^{\left(1\right)}\left(t\right)\right)\\ \;+a_{n-r,n-s}\left(t\right)f_{j}^{\left(2\right)}\left(x_{j}^{\left(2\right)}\left(t\right)\right)+J_{i}^{\left(2\right)}\left(t\right). \end{array}$$

where $x_{i}(t)=(x_{i}^{\left(1\right)}(t),x_{i}^{\left(2\right)}(t)){)}^{T}$ with $x_{i}^{\left(1\right)}\left(t\right)\in R^{r},x_{i}^{\left(2\right)}\left(t\right)\in R^{n-r}$, $f_{j}\left(x_{j}\left(t\right)\right)={\left(f_{j}^{\left(1\right)}\left(x_{j}^{\left(1\right)}\left(t\right)\right),f_{j}^{\left(2\right)}\left(x_{j}^{\left(2\right)}\left(t\right)\right)\right)}^{T}$ with $f_{j}^{\left(1\right)}\left(x_{j}^{\left(1\right)}\left(t\right)\right)\in R^{r},f_{j}^{\left(2\right)}\left(x_{j}^{\left(2\right)}\left(t\right)\right)\in R^{n-r}$, and $J_{i}\left(t\right)={\left(J_{i}^{\left(1\right)}\left(t\right),J_{i}^{\left(2\right)}\left(t\right)\right)}^{T}$.

Models (2) and (3) are equivalent to model (1) with explicit constraints when viewed as a whole. Evidently, model (2) is still a differential system, and a class of AEs constrains model (3). AEs are also nonlinear terms. Thus, DAARTNN is created by merging models (2) and (3).

**Remark 2.1** Obviously, when *rank*(*E*) = 0, the singular ARTNN is an ordinary ARTNN. Here, we assume that 0 < *rank*(*E*) = *r* < *n* is held only to show that the methods and conclusions are also applicable to general NN models.

The coefficient matrices of models 2 and 3 are of different dimensions, but in this paper, we assume that both the DEs and the AEs are in the same dimension. Therefore, with a combined model (2) and (3), we can obtain DAARTNN as follows


(4) $$\{\begin{array}{c} \frac{dx_{i}(t)}{dt}=-c_{i}(t)x_{i}(t)+\sum_{j=1}^{n}a_{ij}(t)f_{j}(x_{j}(t))\\ +\sum_{j=1}^{n}b_{ij}(t)g_{j}(y_{j}(t))+J_{i}\\ 0=-d_{i}(t)y_{i}(t)+\sum_{j=1}^{n}p_{ij}(t)h_{j}(x_{j}(t))\\ +\sum_{j=1}^{n}q_{ij}(t)k_{j}(y_{j}(t))+I_{i} \end{array}$$

where *c*_*i*_(*t*), *d*_*i*_(*t*), *a*_*ij*_(*t*), *b*_*ij*_(*t*), *p*_*ij*_(*t*), and *q*_*ij*_(*t*) are piece-wised continuous and bounded. *f*_*j*_(*), *g*_*j*_(*), *h*_*j*_(*), and *k*_*j*_(*) satisfy


(5) $$\begin{array}{l} 0\leq \frac{f_{j}\left(u\right)-f_{j}\left(v\right)}{u-v}\leq F_{j},0\leq \frac{g_{j}\left(u\right)-g_{j}\left(v\right)}{u-v}\leq G_{j}\\ 0\leq \frac{h_{j}\left(u\right)-h_{j}\left(v\right)}{u-v}\leq H_{j},0\leq \frac{k_{j}\left(u\right)-k_{j}\left(v\right)}{u-v}\leq K_{j}. \end{array}$$

for all *x* ≠ *y*, where *F*_*j*_ > 0, *G*_*j*_ > 0, *H*_*j*_ > 0, and *K*_*j*_ > 0 are all constants and *j* = 1, …, *n*.

Under the strategy of centralized data sampling, the continuous time variable *t* is replaced by a set of discrete sampling time variable *t*_*k*_. After that, the model (4) can be rewritten as


(6) $$\{\begin{array}{c} \frac{dx_{i}(t)}{dt}=-c_{i}(t)x_{i}(t_{k})+\sum_{j=1}^{n}a_{ij}(t)f_{j}(x_{j}(t_{k}))\\ +\sum_{j=1}^{n}b_{ij}(t)g_{j}(y_{j}(t_{k}))+J_{i}\\ 0=-d_{i}(t)y_{i}(t_{k})+\sum_{j=1}^{n}p_{ij}(t)h_{j}(x_{j}(t_{k}))\\ +\sum_{j=1}^{n}q_{ij}(t)k_{j}(y_{j}(t_{k}))+I_{i} \end{array}$$

for *j* = 1, …, *n*. ${\left\{\begin{array}{c} t_{k} \end{array}\right\}}_{k=0}^{+\infty }$ is an increasing time sequence. At each time point *t*_*k*_, all neurons broadcast their state to out-neighbors to receive the state information sent by in-neighbors.

Similarly, under the strategy of decentralized data-sampling, the system (1) can be rewritten as follows:


(7) $$\{\begin{array}{c} \frac{dx_{i}(t)}{dt}=-c_{i}(t)x_{i}(t_{k}^{i})+\sum_{j=1}^{n}a_{ij}(t)f_{j}(x_{j}(t_{k}^{j}))\\ +\sum_{j=1}^{n}b_{ij}(t)g_{j}(y_{j}(t_{k}^{j}))+J_{i}\\ 0=-d_{i}(t)y_{i}(t_{k}^{i})+\sum_{j=1}^{n}p_{ij}(t)h_{j}(x_{j}(t_{k}^{j}))\\ +\sum_{j=1}^{n}q_{ij}(t)k_{j}(y_{j}(t_{k}^{j}))+I_{i} \end{array}$$

for *j* = 1, …, *n*. The increasing time sequence ${\left\{\begin{array}{c} t_{k}^{i} \end{array}\right\}}_{k=0}^{+\infty }$ ordered as $0=t_{0}^{i}<t_{1}^{i}<\cdots <t_{k}^{i}<\cdots$ is uniform for all the neuron $i\in \left\{\begin{array}{c} 1,\ldots ,n \end{array}\right\}$. Each neuron broadcasts its state to its out-neighbors and receives the state information sent by in-neighbors at time $t_{k}^{i}$.

**Remark 2.2** For purposes of sampling-data control, the system only updates its information at periods *t*_*k*_ and $t_{k}^{i}$. The distinction between *t*_*k*_ and $t_{k}^{i}$ is that *t*_*k*_ is centralized whereas $t_{k}^{i}$ is decentralized. There is a variance in the time at which the information is updated. In the centralized style, information is transferred at a specific time point *t*_1_, *t*_2_, *t*_3_, …, *t*_*k*_, …, but in the decentralized style, information is transferred at a specific time point $t_{1}^{1},t_{1}^{2},\ldots ,t_{1}^{n},t_{2}^{1},t_{2}^{2},\cdots ,t_{2}^{n},\cdots ,t_{k}^{1},t_{k}^{2},\cdots$.

To begin the discussion, we give the following definitions and lemmas.

**Definition 1**. There exist a positive constant ς_*i*_, (*i* = 1, …, *n*), the *l*_1_ norm is defined as


$$||x|{|}_{1,\xi }=\overset{n}{\underset{j=1}{\sum }}\varsigma_{i}|x_{i}|.$$

**Definition 2**. Consider any two trajectories (*x*(*t*), *y*(*t*)), (*u*(*t*), *v*(*t*)) of system (4) which starts from different initial values (*x*(0), *y*(0)) and (*u*(0), *v*(0)). The system (4) is said to achieve outer-synchronization if


$$\underset{t\to +\infty }{\lim}||x\left(t\right)-u\left(t\right)||=0,\underset{t\to +\infty }{\lim}||y\left(t\right)-v\left(t\right)||=0,$$

where ||·|| is the norm of state.

We aimed to transform DAS to the form of regular differential equations by differential operations since they are a combination of differential equations and AEs. The index of the DAS is the quantity of differentials employed in this procedure. For instance, a differential equation is index-0.

**Lemma 1**. The DAARTNN is said to be index-1, if and only if


$$-d_{i}\left(t\right)+\overset{n}{\underset{j=1}{\sum }}p_{ij}\left(t\right)h_{j}^{\prime }\left(x_{j}\left(t\right)\right)+\overset{n}{\underset{j=1}{\sum }}q_{ij}\left(x_{j}\left(t\right)\right)k_{j}^{\prime }\left(y_{j}\left(t\right)\right)>0$$

Based the research, we set


$$\begin{array}{l} \mu_{j}\left(\xi ,t\right)=c_{j}\left(t\right)-F_{j}a_{jj}^{+}\left(t\right)-F_{j}\underset{i\neq j}{\sum }\frac{\varsigma_{i}}{\varsigma_{j}}|a_{ij}\left(t\right)|,\\ v_{j}\left(\xi ,t\right)=G_{j}b_{jj}^{+}\left(t\right)+G_{j}\underset{i\neq j}{\sum }\frac{\varsigma_{i}}{\varsigma_{j}}|b_{ij}\left(t\right)|,\\ \sigma_{j}\left(t\right)=\frac{p_{jj}^{+}\left(t\right)H_{j}+H_{j}\underset{i\neq j}{\sum }|p_{ij}\left(t\right)|}{d_{j}\left(t\right)-q_{jj}^{+}\left(t\right)-K_{j}\underset{i\neq j}{\sum }|q_{ij}\left(t\right)|},\\ M_{1}=\underset{i\leq j\leq n}{\max}\underset{t\geq t_{0}}{\sup}\left\{c_{j}\left(t\right)+F_{j}a_{jj}^{+}\left(t\right)+F_{j}\underset{j\neq i}{\sum }\frac{\varsigma_{i}}{\varsigma_{j}}|a_{ij}\left(t\right)|\right\},\\ M_{2}=\underset{i\leq j\leq n}{\max}\underset{t\geq t_{0}}{\sup}\left\{G_{j}b_{jj}^{+}\left(t\right)+G_{j}\underset{i\neq j}{\sum }\frac{\varsigma_{i}}{\varsigma_{j}}|b_{ij}\left(t\right)|\right\}, \end{array}$$

where $a_{ii}^{+}\left(t\right)=\max\left\{a_{ii}\left(t\right),0\right\},$ $b_{ii}^{+}\left(t\right)=\max\left\{b_{ii}\left(t\right),0\right\},$ $a_{ii}^{-}\left(t\right)=\min\left\{a_{ii}\left(t\right),0\right\},$ and $b_{ii}^{-}\left(t\right)=\min\left\{b_{ii}\left(t\right),0\right\}.$

Because *v*_*j*_(ξ, *t*), δ_*j*_(*t*) are bounded, this means that there is a constant δ satisfying the following conditions


$$\begin{array}{l} \underset{t\in \left[0,+\infty \right)}{\sup}\sigma_{j}\left(t\right)\leq \delta ,\\ \sup\left\{\mu_{j}\left(\xi ,s\right)-\delta v_{j}\left(\xi ,s\right)\right\}\leq N. \end{array}$$

## 3. Main results

This section shows how to build the controls of the system using the settings from the previous section and the centralized and decentralized sampling of data principles.

### 3.1. Structure-dependent centralized and decentralized data sampling

Denote $w\left(t\right)={\left[w_{1}\left(t\right),\ldots ,w_{n}\left(t\right)\right]}^{T}$ and $z\left(t\right)={\left[z_{1}\left(t\right),\ldots ,z_{n}\left(t\right)\right]}^{T}$ with *w*_*i*_(*t*) = *x*_*i*_(*t*) − *u*_*i*_(*t*), *z*_*i*_(*t*) = *y*_*i*_(*t*) − *v*_*i*_(*t*), and ${\bar{f}}_{i}\left(t\right)=f_{i}\left(x_{i}\left(t\right)\right)-f_{i}\left(u_{i}\left(t\right)\right),{ḡ}_{i}\left(t\right)=g_{i}\left(y_{i}\left(t\right)\right)-g_{i}\left(v_{i}\left(t\right)\right),{\bar{h}}_{i}\left(t\right)=h_{i}\left(x_{i}\left(t\right)\right)-h_{i}\left(u_{i}\left(t\right)\right),{\bar{k}}_{i}\left(t\right)=k_{i}\left(y_{i}\left(t\right)\right)-k_{i}\left(v_{i}\left(t\right)\right)$. Then it holds


(8) $$\{\begin{array}{c} \frac{dw_{i}(t)}{dt}=-c_{i}(t)w_{i}(t_{k})+\sum_{j=1}^{n}a_{ij}(t){\bar{f}}_{j}(t_{k})+\sum_{j=1}^{n}b_{ij}(t){\bar{g}}_{j}(t_{k})\\ 0=-d_{i}(t)z_{i}(t_{k})+\sum_{j=1}^{n}p_{ij}(t){\bar{h}}_{j}(t_{k})+\sum_{j=1}^{n}q_{ij}(t){\bar{k}}_{j}(t_{k}), \end{array}$$

for all *t* ∈ [*t*_*k*_, *t*_*k*+1_), *i* = 1, …, *n* and *k* = 0, 1, 2, ….

The following theorem gives conditions that guarantee the system (4) reaches outer-synchronization *via* *l*_1_−norm.

**Theorem 1**. Assume that ε_*a*_ ∈ (0, 1) and ε_0_ > 0 with *Nε*_*a*_ ≤ ε_0_(2 − ε_*a*_). Suppose μ_*j*_(ξ, *s*) − δ*v*_*j*_(ξ, *s*) ≥ ε_0_. Set an increasing time-point sequence {*t*_*k*_} as


(9) $$\begin{array}{l} t_{k+1}=\underset{\tau \geq t_{k}}{\sup}\{\tau :\underset{j=1,\ldots ,n}{\min}\int_{t_{k}}^{t}[\mu_{j}\left(\xi ,s\right)-\\ \;\delta v_{j}(\xi ,s)]ds\leq \varepsilon_{a},\forall t\in (t_{k},\tau ]\}. \end{array}$$

**Proof**. Since μ_*j*_(ξ, *s*) − δ*v*_*j*_(ξ, *s*) ≥ ε_0_ and the positive upper bound of it, we have


(10) $$\begin{array}{l} \varepsilon_{0}\left(t-t_{k}\right)\leq \int_{t_{k}}^{t}\left[\mu_{j}\left(\xi ,s\right)-\delta v_{j}\left(\xi ,s\right)\right]ds\leq N\left(t-t_{k}\right), \end{array}$$

where *j* = 1, 2, …, *n* and *t* ∈ [*t*_*k*_, *t*_*k*+1_]. Based on data-sampling principles (9), the state will not be sampled until the following equation holds:


(11) $$\begin{array}{l} \int_{t_{k}}^{t}\left[\mu_{j}\left(\xi ,s\right)-\delta v_{j}\left(\xi ,s\right)\right]ds=\varepsilon_{a}, \end{array}$$

when *t* = *t*_*k*+1_, from (10) and (11),


$$\begin{array}{l} \varepsilon_{0}\left(t_{k+1}-t_{k}\right)\leq \varepsilon_{a}\leq N\left(t_{k+1}-t_{k}\right), \end{array}$$

then


(12) $$\begin{array}{l} \frac{\varepsilon_{a}}{N}\leq t_{k+1}-t_{k}\leq \frac{\varepsilon_{a}}{\varepsilon_{0}}, \end{array}$$

so,


(13) $$\begin{array}{l} \int_{t_{k}}^{t_{k+1}}\left[\mu_{j}\left(\xi ,s\right)-\delta v_{j}\left(\xi ,s\right)\right]ds\leq N\left(t_{k+1}-t_{k}\right)\leq \frac{N\varepsilon_{a}}{\varepsilon_{0}}, \end{array}$$

combined (12) and (13), we have


(14) $$\begin{array}{l} \varepsilon_{a}\leq \int_{t_{k}}^{t_{k+1}}\left[\mu_{j}\left(\xi ,s\right)-\delta v_{j}\left(\xi ,s\right)\right]ds\leq 2-\varepsilon_{a}. \end{array}$$

Consider *w*_*i*_(*t*)(*i* = 1, …, *n*) for each *t* ∈ [*t*_*k*_, *t*_*k*+1_], we have


(15) $$\begin{array}{l} \;\overset{n}{\underset{i=1}{\sum }}\varsigma_{i}|w_{i}\left(t\right)|\\ =\overset{n}{\underset{i=1}{\sum }}\varsigma_{i}|w_{i}\left(t_{k}\right)+\int_{t_{k}}^{t}{ẇ}_{i}\left(s\right)ds|\\ =\overset{n}{\underset{i=1}{\sum }}|\varsigma_{i}w_{i}\left(t_{k}\right)-\int_{t_{k}}^{t}\left[c_{i}\left(s\right)-a_{ii}\left(s\right)m_{i}\left(s\right)\right]ds\varsigma_{i}w_{i}\left(t_{k}\right)\\ \;+\int_{t_{k}}^{t}\left[b_{ii}\left(s\right)n_{i}\left(s\right)\right]ds\varsigma_{i}z_{i}\left(t_{k}\right)+\underset{j\neq i}{\sum }\int_{t_{k}}^{t}\left[a_{ij}\left(s\right)\frac{\varsigma_{i}}{\varsigma_{j}}m_{j}\left(t_{k}\right)\right]ds\\ \;\varsigma_{j}w_{j}\left(t_{k}\right)+\underset{j\neq i}{\sum }\int_{t_{k}}^{t}\left[b_{ij}\left(s\right)\frac{\varsigma_{i}}{\varsigma_{j}}n_{j}\left(t_{k}\right)\right]ds\varsigma_{j}z_{j}\left(t_{k}\right)|, \end{array}$$

with


(16) $$m_{j}(t)=\{\begin{array}{cc} \frac{{\bar{f}}_{j}(t)}{w_{j}(t)}, & w_{j}(t)\neq 0\\ 0, & w_{j}(t)=0 \end{array}$$

(17) $$n_{j}(t)=\{\begin{array}{cc} \frac{{\bar{g}}_{j}(t)}{z_{j}(t)}, & z_{j}(t)\neq 0\\ 0, & z_{j}(t)=0 \end{array}$$

which implies 0 ≤ *m*_*j*_(*t*) ≤ *F*_*j*_, 0 ≤ *n*_*j*_(*t*) ≤ *G*_*j*_ for all *j* = 1, …, *n* and *k* = 0, 1, …, from above, note


$$\begin{array}{l} {\left(a_{ii}\left(s\right)\right)}^{-}F_{i}\leq a_{ii}\left(s\right)m_{i}\left(t\right)\leq {\left(a_{ii}\left(s\right)\right)}^{+}F_{i}\\ {\left(b_{ii}\left(s\right)\right)}^{-}G_{i}\leq b_{ii}\left(s\right)n_{i}\left(t\right)\leq {\left(b_{ii}\left(s\right)\right)}^{+}G_{i}. \end{array}$$

Then, it follows


(18) $$\begin{array}{l} \overset{n}{\underset{i=1}{\sum }}\varsigma_{i}|w_{i}\left(t\right)|\\ \leq \overset{n}{\underset{j=1}{\sum }}\{|1-\int_{t_{k}}^{t}\left[c_{j}\left(s\right)-a_{jj}^{+}\left(s\right)F_{j}\right]ds\\ +\underset{j\neq i}{\sum }\frac{\varsigma_{i}}{\varsigma_{j}}\int_{t_{k}}^{t}\left[|a_{ij}\left(s\right)|F_{j}\right]ds|\}\varsigma_{j}|w_{j}\left(t_{k}\right)|\\ +\overset{n}{\underset{j=1}{\sum }}\{|\int_{t_{k}}^{t}\left[b_{jj}^{+}\left(s\right)G_{j}\right]ds\\ +\underset{j\neq i}{\sum }\frac{\varsigma_{i}}{\varsigma_{j}}\int_{t_{k}}^{t}\left[|b_{ij}\left(s\right)|G_{j}\right]ds|\}\varsigma_{j}|z_{j}\left(t_{k}\right)|. \end{array}$$

For static equation


(19) $$\begin{array}{l} d_{i}\left(t\right)z_{i}\left(t_{k}\right)=p_{ii}\left(t\right){\bar{h}}_{i}\left(t_{k}\right)+q_{ii}\left(t\right){\bar{k}}_{i}\left(t_{k}\right)\\ \;+\underset{j\neq i}{\sum }\left(p_{ij}\left(t\right){\bar{h}}_{j}\left(t_{k}\right)+q_{ij}\left(t\right){\bar{k}}_{j}\left(t_{k}\right)\right)\\ d_{j}\left(t\right)z_{j}\left(t_{k}\right)=p_{jj}\left(t\right)r_{j}\left(t_{k}\right)w_{j}\left(t_{k}\right)+q_{jj}\left(t\right)s_{j}\left(t_{k}\right)z_{j}\left(t_{k}\right)\\ \;+\underset{j\neq i}{\sum }\left(p_{ij}\left(t\right)r_{j}\left(t_{k}\right)w_{j}\left(t_{k}\right)+q_{ij}\left(t\right)s_{j}\left(t_{k}\right)z_{j}\left(t_{k}\right)\right), \end{array}$$

with


(20) $$r_{j}(t)=\{\begin{array}{cc} \frac{{\bar{h}}_{j}(t)}{w_{j}(t)}, & w_{j}(t)\neq 0\\ 0, & w_{j}(t)=0 \end{array}$$

(21) $$s_{j}(t)=\{\begin{array}{cc} \frac{{\bar{k}}_{j}(t)}{z_{j}(t)}, & z_{j}(t)\neq 0\\ 0, & z_{j}(t)=0. \end{array}$$

From above, we have


(22) $$\begin{array}{l} \left[d_{j}(t)-q_{jj}(t)s_{j}(t_{k})-\sum_{j\neq i}q_{ij}(t)s_{j}(t_{k})]z_{j}(t_{k}\right)\\ \;=[p_{jj}(t)r_{j}(t_{k})+\sum_{j\neq i}p_{ij}(t)r_{j}(t_{k})]w_{j}(t_{k}), \end{array}$$

we can obtain


(23) $$\begin{array}{l} z_{j}\left(t_{k}\right)=\frac{p_{jj}\left(t\right)r_{j}\left(t_{k}\right)+\underset{j\neq i}{\sum }p_{ij}\left(t\right)r_{j}\left(t_{k}\right)}{d_{j}\left(t\right)-q_{jj}\left(t\right)s_{j}\left(t_{k}\right)-\underset{j\neq i}{\sum }q_{ij}\left(t\right)s_{j}\left(t_{k}\right)}w_{j}\left(t_{k}\right). \end{array}$$

Note that


$$\begin{array}{l} {\left(p_{ii}\left(s\right)\right)}^{-}H_{i}\leq p_{ii}\left(s\right)m_{i}\left(t_{k}\right)\leq {\left(p_{ii}\left(s\right)\right)}^{+}H_{i}\\ {\left(q_{ii}\left(s\right)\right)}^{-}K_{i}\leq q_{ii}\left(s\right)n_{i}\left(t_{k}\right)\leq {\left(q_{ii}\left(s\right)\right)}^{+}K_{i}, \end{array}$$

thus we can get


(24) $$\begin{array}{l} z_{j}\left(t_{k}\right)\leq \sigma_{j}\left(t\right)w_{j}\left(t_{k}\right), \end{array}$$

where


(25) $$\begin{array}{l} \sigma_{j}\left(t\right)=\frac{p_{jj}\left(t\right)r_{j}\left(t_{k}\right)+\underset{j\neq i}{\sum }p_{ij}\left(t\right)r_{j}\left(t_{k}\right)}{d_{j}\left(t\right)-q_{jj}\left(t\right)s_{j}\left(t_{k}\right)-\underset{j\neq i}{\sum }q_{ij}\left(t\right)s_{j}\left(t_{k}\right)}. \end{array}$$

Combining (18) and (25), we can observe


(26) $$\begin{array}{l} \;\overset{n}{\underset{i=1}{\sum }}\varsigma_{i}|w_{i}\left(t\right)|\\ \leq \overset{n}{\underset{j=1}{\sum }}\{|1-\int_{t_{k}}^{t}\left[c_{j}\left(s\right)-a_{jj}^{+}\left(s\right)F_{j}\right]ds\\ \;+\underset{j\neq i}{\sum }\frac{\varsigma_{i}}{\varsigma_{j}}\int_{t_{k}}^{t}\left[|a_{ij}\left(s\right)|F_{j}\right]ds|\}\varsigma_{j}|w_{j}\left(t_{k}\right)|\\ \;+\overset{n}{\underset{j=1}{\sum }}\left\{|\int_{t_{k}}^{t}\left[b_{jj}^{+}\left(s\right)G_{j}\right]ds+\underset{j\neq i}{\sum }\frac{\varsigma_{i}}{\varsigma_{j}}\int_{t_{k}}^{t}\left[|b_{ij}\left(s\right)|G_{j}\right]ds|\right\}\\ \;\sigma_{j}\left(t\right)\varsigma_{j}|w_{j}\left(t_{k}\right)\\ \leq \overset{n}{\underset{j=1}{\sum }}|1-\int_{t_{k}}^{t}\left[\mu_{j}\left(\xi ,s\right)ds-\delta v_{j}\left(\xi ,s\right)\right]ds|\varsigma_{j}|w_{j}\left(t_{k}\right)|, \end{array}$$

since the equality (9) occurs at *t* = *t*_*k*+1_, thus we have


$$\overset{n}{\underset{i=n}{\sum }}\varsigma_{i}|w_{i}\left(t_{k+1}\right)|\leq \left(1-\varepsilon_{a}\right)\overset{n}{\underset{i=n}{\sum }}\varsigma_{i}|w_{i}\left(t_{k}\right)|,$$

which implies


$$\underset{t\to +\infty }{\lim}||w\left(t_{k}\right)|{|}_{1,\xi }=0.$$

In addition, for each *t* ∈ (*t*_*k*_, *t*_*k*+1_), from the rule (9), the inequality (16) implies that ||*w*(*t*)||_1,ξ_ ≤ ||*w*(*t*_*k*_)||_1,ξ_. Hence, it holds


$$\underset{t\to +\infty }{\lim}||w\left(t\right)|{|}_{1,\xi }=0,$$

then from condition (11), we have


$$\underset{t\to +\infty }{\lim}||z\left(t\right)|{|}_{1,\xi }=0.$$

The out-synchronization of the system (4) is proved.

**Remark 3.1**. The sampling interval is positive. Each interval has a common positive lower bound based on (12). This result avoids the Zeno phenomenon during sampling.

Under decentralized principles, this section will consider the system below


(27) $$\{\begin{array}{c} \frac{dw_{i}(t)}{dt}=-c_{i}(t)w_{i}(t_{k}^{i})+\sum_{j=1}^{n}a_{ij}(t){\bar{f}}_{j}(t_{k}^{j})+\sum_{j=1}^{n}b_{ij}(t){\bar{g}}_{j}(t_{k}^{j})\\ 0=-d_{i}(t)z_{i}(t_{k}^{i})+\sum_{j=1}^{n}p_{ij}(t){\bar{h}}_{j}(t_{k}^{j})+\sum_{j=1}^{n}q_{ij}(t){\bar{k}}_{j}(t_{k}^{j}), \end{array}$$

for all $t\in \left[t_{k}^{j},t_{k+1}^{j}\right),$ *i* = 1, …, *n* and *k* = 0, 1, 2, ⋯ .

**Remark 3.2**. To further illustrate the mechanism of decentralizing the sampling data, let *l*_*k*_ be the time point at which events are updated across the network. Then *l*_*k*_ are satisfied as follows


$${\left\{l_{k}\right\}}_{k=0}^{+\infty }=\overset{+\infty }{\underset{k=0}{⋃}}\overset{n}{\underset{i=1}{⋃}}t_{k}^{i},$$

The following theorem gives conditions that guarantee the convergence of system (27) *via* *l*_1_ norm.

**Theorem 2**. Let ε_*b*_ ∈ (0, 1), ε_0_ > 0, and *Nε*_*b*_ ≤ ε_0_ set at a time point *t*_*k*_, the state will renew information until the following condition holds:


(28) $$\begin{array}{l} t_{k+1}^{i}=\underset{\tau \geq t_{k}^{i}}{\sup}\{\tau :\underset{j=1,\ldots ,n}{\min}\int_{t_{k}^{i}}^{t}[\mu_{j}(\xi ,s)-\\ \;\delta v_{j}(\xi ,s)]ds\leq \varepsilon_{b},\forall t\in (t_{k}^{i},\tau ]\} \end{array}$$

for *i* = 1, 2, …, *n* and *k* = 0, 1, 2, ⋯ , then the condition guarantees the system (4) to reach outer-synchronization.

**Proof**. Just similar to (12)


(29) $$\begin{array}{l} \frac{\varepsilon_{b}}{N}\leq t_{k+1}^{i}-t_{k}^{i}\leq \frac{\varepsilon_{b}}{\varepsilon_{0}}, \end{array}$$

(30) $$\begin{array}{l} \varepsilon_{b}\leq \int_{t_{k}^{i}}^{t_{k+1}^{i}}\left[\mu_{j}\left(\xi ,s\right)-\delta v_{j}\left(\xi ,s\right)\right]ds\leq N\left(t_{k+1}^{i}-t_{k}^{i}\right)\\ \leq \frac{N\varepsilon_{b}}{\varepsilon_{0}}\leq 1. \end{array}$$

Consider *w*_*i*_(*t*) for any neuron *i* at triggering time $t_{k+1}^{i}$, where *i* = 1, …, *n*, we have


(31) $$\begin{array}{l} \;\sum_{i=1}^{n}\varsigma_{i}|w_{i}(t_{k+1}^{i})|\\ =\sum_{i=1}^{n}sign(w_{i}(t_{k+1}^{i}))\varsigma_{i}[w_{i}(t_{k}^{i})+\int_{t_{k}^{i}}^{t_{k+1}^{i}}{\dot{w}}_{i}(s)ds],\\ =\sum_{i=1}^{n}sign(w_{i}(t_{k+1}^{i}))\varsigma_{i}w_{i}(t_{k}^{i})-\sum_{i=1}^{n}sign(w_{i}(t_{k+1}^{i}))\\ \;\varsigma_{i}\int_{t_{k}^{i}}^{t_{k+1}^{i}}[-c_{i}w_{i}(t_{k}^{i})+\sum_{j=1}^{n}a_{ij}(s){\bar{f}}_{j}(w(t_{k}^{j}))\\ \;+\sum_{j=1}^{n}b_{ij}(s){\bar{g}}_{j}(z(t_{k}^{j}))]ds,\\ =\sum_{i=1}^{n}sign(w_{i}(t_{k+1}^{i}))\varsigma_{i}w_{i}(t_{k}^{i})\\ \;-\sum_{i=1}^{n}sign(w_{i}(t_{k+1}^{i}))\varsigma_{i}w_{i}(t_{k}^{i})\int_{t_{k}^{i}}^{t_{k+1}^{i}}c_{i}ds\\ \;+\sum_{i=1}^{n}sign(w_{i}(t_{k+1}^{i}))\varsigma_{i}{\bar{f}}_{i}(w(t_{k}^{j}))\int_{t_{k}^{i}}^{t_{k+1}^{i}}a_{ii}^{+}(s)ds\\ \;+\sum_{i=1}^{n}sign(w_{i}(t_{k+1}^{i}))\varsigma_{i}{\bar{g}}_{i}(z(t_{k}^{j}))\int_{t_{k}^{i}}^{t_{k+1}^{i}}b_{ii}^{+}(s)ds\\ \;+\sum_{j\neq i}sign(w_{i}(t_{k+1}^{i}))\varsigma_{i}\sum_{i=1}^{n}{\bar{f}}_{j}(w(t_{k}^{j}))\int_{t_{k}^{i}}^{t_{k+1}^{i}}a_{ij}^{+}(s)ds\\ \;+\sum_{j\neq i}sign(w_{i}(t_{k+1}^{i}))\varsigma_{i}\sum_{j=1}^{n}{\bar{g}}_{j}(z(t_{k}^{j}))\int_{t_{k}^{i}}^{t_{k+1}^{i}}b_{ij}^{+}(s)ds, \end{array}$$

then, it holds


(32) $$\begin{array}{l} \;\overset{n}{\underset{i=1}{\sum }}\varsigma_{i}|w_{i}\left(t_{k+1}^{i}\right)|\\ \leq \overset{n}{\underset{j=1}{\sum }}\{1-\int_{t_{k}^{j}}^{t_{k+1}^{j}}\left[c_{j}-F_{j}a_{jj}^{+}\left(s\right)\right]ds\\ \;+F_{j}\underset{j\neq i}{\sum }\frac{\varsigma_{i}}{\varsigma_{j}}\int_{t_{k}^{j}}^{t_{k+1}^{j}}|a_{ij}\left(s\right)|ds\}\varsigma_{j}|w_{j}\left(t_{k}^{j}\right)|\\ \;+\overset{n}{\underset{j=1}{\sum }}\{\int_{t_{k}^{j}}^{t_{k+1}^{j}}G_{j}b_{jj}^{+}\left(s\right)ds\\ \;+G_{j}\underset{j\neq i}{\sum }\frac{\varsigma_{i}}{\varsigma_{j}}\int_{t_{k}^{i}}^{t_{k+1}^{i}}|b_{ij}\left(s\right)|ds\}\varsigma_{j}|z_{j}\left(t_{k}^{j}\right)|. \end{array}$$

From static equation of system (27), we have


(33) $$\begin{array}{l} z_{j}\left(t_{k}^{j}\right)=\frac{p_{jj}\left(t\right)r_{j}\left(t_{k}^{j}\right)+\underset{j\neq i}{\sum }p_{ij}\left(t\right)r_{j}\left(t_{k}^{j}\right)}{d_{j}-q_{jj}\left(t\right)s_{j}\left(t_{k}^{j}\right)-\underset{j\neq i}{\sum }q_{ij}\left(t\right)s_{j}\left(t_{k}^{j}\right)}w_{j}\left(t_{k}^{j}\right)\\ \;\leq \frac{p_{jj}^{+}\left(t\right)H_{j}+\underset{j\neq i}{\sum }|p_{ij}\left(t\right)|H_{j}}{d_{j}-q_{jj}^{+}\left(t\right)K_{j}-\underset{j\neq i}{\sum }|q_{ij}\left(t\right)|K_{j}}w_{j}\left(t_{k}^{j}\right)\\ \;=\sigma_{j}\left(t\right)w_{j}\left(t_{k}^{j}\right). \end{array}$$

Then, combining (32) and (33) we can get


(34) $$\begin{array}{l} \;\sum_{i=1}^{n}\varsigma_{i}|w_{i}(t_{k+1}^{i})|\\ \leq \{\sum_{j=1}^{n}\{1-\int_{t_{k}^{j}}^{t_{k+1}^{j}}[c_{j}-F_{j}a_{jj}^{+}(s)]ds\\ \;+F_{j}\sum_{j\neq i}\frac{\varsigma_{i}}{\varsigma_{j}}\int_{t_{k}^{i}}^{t_{k+1}^{i}}|a_{ij}(s)|ds\}\\ \;+\sum_{j=1}^{n}\{\int_{t_{k}^{j}}^{t_{k+1}^{j}}G_{j}b_{jj}^{+}(s)ds\\ \;+G_{j}\sum_{j\neq i}\frac{\varsigma_{i}}{\varsigma_{j}}\int_{t_{k}^{i}}^{t_{k+1}^{i}}|b_{ij}(s)|\}\sigma_{j}(t)\}\varsigma_{j}w_{j}(t_{k}^{j})\\ \leq (1-\varepsilon_{b})\varsigma_{j}w_{j}(t_{k}^{j}). \end{array}$$

Based the triggering rule (28), we can obtain


(35) $$\begin{array}{l} \overset{n}{\underset{i=1}{\sum }}\varsigma_{i}|w_{i}\left(t_{k+1}^{i}\right)|\leq \left(1-\varepsilon_{b}\right)\varsigma_{j}w_{j}\left(t_{k}^{j}\right), \end{array}$$

which means


$$\underset{t_{k}^{i}\to +\infty }{\lim}||w\left(t_{k}^{i}\right)|{|}_{1}=0.$$

For any time $t\in \left(t_{k}^{i},t_{k+1}^{i}\right],$ the state *w*_*i*_(*t*) becomes


(36) $$\begin{array}{l} \;\sum_{i=1}^{n}\varsigma_{i}|w_{i}(t)|\\ \leq \sum_{j=1}^{n}\{1-\int_{t_{k}^{j}}^{t}[c_{j}-a_{jj}^{+}(s)F_{j}]ds\\ \;+F_{j}\sum_{j\neq i}\frac{\varsigma_{i}}{\varsigma_{j}}\int_{t_{k}^{i}}^{t}|a_{ij}(s)|ds+\sigma_{j}(t)G_{j}\int_{t_{k}^{j}}^{t}b_{jj}^{+}(s)ds\\ \;+\sigma_{j}(t)G_{j}\sum_{j\neq i}\frac{\varsigma_{i}}{\varsigma_{j}}\int_{t_{k}^{j}}^{t}b_{jj}^{+}(s)ds\}\varsigma_{j}|w_{j}(t_{k})|\\ \leq \sum_{j=1}^{n}\{1-\int_{t_{k}^{i}}^{t}[c_{j}-a_{jj}^{+}(s)F_{j}]ds\\ \;+F_{j}\sum_{j\neq i}\frac{\varsigma_{i}}{\varsigma_{j}}[\int_{t_{k}^{i}}^{t}|a_{ij}(s)|ds+\int_{t}^{\tau }|a_{ij}(s)|ds]\\ \;+\sigma_{j}(t)G_{j}\int_{t_{k}^{j}}^{t}b_{jj}^{+}(s)ds\\ \;+\sigma_{j}(t)G_{j}\sum_{j\neq i}\frac{\varsigma_{i}}{\varsigma_{j}}[\int_{t_{k}^{j}}^{t}b_{jj}^{+}(s)ds\\ \;+\int_{t}^{\tau }|b_{ij}(s)|ds]\}\varsigma_{j}|w_{j}(t_{k})|\\ \leq (1-\varepsilon_{b})\|w(t_{k}^{i})\| \end{array}$$

where $t_{k+1}^{i}\geq \tau >t>t_{k}^{i}$. Thus


$$||w\left(t_{k+1}^{i}\right)|{|}_{1}\leq ||w\left(t\right)|{|}_{1}\leq \left(1-\varepsilon_{b}\right)||w\left(t_{k}^{i}\right)|{|}_{1},$$

for any $t\in \left(t_{k}^{i},t_{k+1}^{i}\right]$ and *i* = 1, …, *n*, which implies


$$\underset{t\to +\infty }{\lim}||w\left(t\right)|{|}_{1}\leq \underset{t_{k}^{i}\to +\infty }{\lim}||w\left(t_{k}^{i}\right)|{|}_{1}=0.$$

The proof for the out-synchronization of the system (4) is completed.

**Remark 3.3**. Theorems 1 and 2 are based on centralized and decentralized data sampling under the system structure, and the research process is extremely dependent on the structural characteristics of the model.

### 3.2. State-dependent centralized and decentralized data sampling

In this section, we established a sampling control mechanism according to the state characteristics of the system. Under this sampling mechanism, neurons transmit and update information at the next triggering time point.

In system (6), the state measurement error is defined as


$$e_{i}\left(t\right)=w_{i}\left(t_{k}\right)-w_{i}\left(t\right),$$

and the state measurement of static equation is as follows:


$$\eta_{i}\left(t\right)=z_{i}\left(t_{k}\right)-z_{i}\left(t\right),$$

where *t* ∈ [*t*_*k*_, *t*_*k*+1_), *i* = 1, …, *n* and *k* = 0, 1, 2, …

**Theorem 3**. Let ℏ(*t*) be a positive decreasing continuous function on [0, +∞) with φ(0) > 0. Set *t*_*k*+1_ as the triggering time point such that


(37) $$\begin{array}{l} t_{k+1}=\underset{ı\geq t_{k}}{\max}\left\{ı:||e\left(t\right)||\leq \hbar \left(t\right),\forall t\in \left(t_{k},ı\right)\right\}, \end{array}$$

for *i* = 1, …, *n* and *k* = 0, 1, 2… There exist ς_*i*_, if μ_*j*_(ξ, *t*) ≥ ε_3_,


$$\underset{t\to +\infty }{\lim}\hbar \left(t\right)=0,$$

then, the system (4) reaches out-synchronization.

**Proof**. Consider *w*_*i*_(*t*) for any neuron *i*(*i* = 1, …, *n*) and ς_*i*_ > 0(*i* = 1, …, *n*)


(38) $$\begin{array}{l} \;\frac{d\|w(t){\|}_{1}}{dt}\\ =\sum_{i=1}^{n}\varsigma_{i}sign(w_{i}(t))\frac{d\|w_{i}(t)\|}{dt}\\ =\sum_{i=1}^{n}\varsigma_{i}sign(w_{i}(t))[-c_{i}w_{i}(t_{k})+\sum_{j=1}^{n}a_{ij}(t){\bar{f}}_{j}(w_{j}(t_{k}))\\ \;+\sum_{j=1}^{n}b_{ij}(t){\bar{g}}_{j}(z_{j}(t_{k}))],\\ =\sum_{i=1}^{n}\varsigma_{i}sign(w_{i}(t))[-c_{i}w_{i}(t)+\sum_{j=1}^{n}a_{ij}(t){\bar{f}}_{j}(w_{j}(t))\\ \;+\sum_{j=1}^{n}b_{ij}(t){\bar{g}}_{j}(z_{j}(t))]+\sum_{i=1}^{n}\varsigma_{i}sign(w_{i}(t))[-c_{i}(w_{i}(t_{k})-w_{i}(t)]\\ \;+\sum_{i=1}^{n}\varsigma_{i}sign(w_{i}(t))\sum_{j=1}^{n}a_{ij}(t)[{\bar{f}}_{j}(w_{j}(t_{k}))-{\bar{f}}_{j}(w_{j}(t))]\\ \;+\sum_{i=1}^{n}\varsigma_{i}sign(w_{i}(t))\sum_{j=1}^{n}b_{ij}(t)[{\bar{g}}_{j}(z_{j}(t_{k}))-{\bar{g}}_{j}(z_{j}(t))], \end{array}$$

from (38), it holds


(39) $$\begin{array}{l} \;\frac{d||w\left(t\right)|{|}_{1}}{dt}\\ =\overset{n}{\underset{i=1}{\sum }}\varsigma_{i}sign\left(w_{i}\left(t\right)\right)\frac{d||w_{i}\left(t\right)||}{dt}\\ \leq -\overset{n}{\underset{i=1}{\sum }}\varsigma_{i}c_{i}|w_{i}\left(t\right)|+\varsigma_{i}c_{i}|e_{i}\left(t\right)|+\overset{n}{\underset{i=1}{\sum }}\varsigma_{i}a_{ii}^{+}\left(t\right)F_{i}|w_{i}\left(t\right)|\\ \;+\overset{n}{\underset{i=1}{\sum }}\varsigma_{i}b_{ii}^{+}\left(t\right)G_{i}|z_{i}\left(t\right)|+\overset{n}{\underset{i=1}{\sum }}\varsigma_{i}a_{ii}^{+}\left(t\right)F_{i}|e_{i}\left(t\right)|\\ \;+\overset{n}{\underset{i=1}{\sum }}\varsigma_{i}b_{ii}^{+}\left(t\right)G_{i}|\eta_{i}\left(t\right)|+\overset{n}{\underset{j=1}{\sum }}\underset{j\neq i}{\sum }\varsigma_{i}|a_{ij}|F_{j}|w_{j}\left(t\right)|\\ \;+\overset{n}{\underset{j=1}{\sum }}\underset{j\neq i}{\sum }\varsigma_{i}|a_{ij}|F_{j}|e_{j}\left(t\right)|+\overset{n}{\underset{j=1}{\sum }}\underset{j\neq i}{\sum }\varsigma_{i}|b_{ij}|G_{j}|z_{j}\left(t\right)|\\ \;+\overset{n}{\underset{j=1}{\sum }}\underset{j\neq i}{\sum }\varsigma_{i}|b_{ij}|G_{j}|\eta_{j}\left(t\right)|,\\ =-\overset{n}{\underset{j=1}{\sum }}\left[c_{j}-F_{j}a_{jj}^{+}\left(t\right)-F_{j}\underset{j\neq i}{\sum }\frac{\varsigma_{i}}{\varsigma_{j}}|a_{ij}\left(t\right)|\right]\varsigma_{j}|w_{j}\left(t\right)|\\ \;+\overset{n}{\underset{j=1}{\sum }}\left[c_{j}+F_{j}a_{jj}^{+}\left(t\right)+\underset{j\neq i}{\sum }\frac{\varsigma_{i}}{\varsigma_{j}}|a_{ij}\left(t\right)|\right]\varsigma_{j}|e_{j}\left(t\right)|\\ \;+\left[G_{j}b_{jj}^{+}\left(t\right)+G_{j}\underset{j\neq i}{\sum }\frac{\varsigma_{i}}{\varsigma_{j}}|b_{ij}\left(t\right)|\right]\varsigma_{j}|z_{j}\left(t\right)|\\ \;+\left[G_{j}b_{jj}^{+}\left(t\right)+G_{j}\underset{j\neq i}{\sum }\frac{\varsigma_{i}}{\varsigma_{j}}|b_{ij}\left(t\right)|\right]\varsigma_{j}|\eta_{j}\left(t\right)|. \end{array}$$

For static equation, we have


(40) $$\begin{array}{l} d_{i}\left(z_{i}\left(t_{k}\right)-z_{i}\left(t\right)\right)=\overset{n}{\underset{j=1}{\sum }}p_{ij}\left(t\right)\left[{\bar{h}}_{j}\left(w_{j}\left(t_{k}\right)\right)-{\bar{h}}_{j}\left(w_{j}\left(t\right)\right)\right]\\ \;+\overset{n}{\underset{j=1}{\sum }}q_{ij}\left(t\right)\left[{\bar{k}}_{j}\left(z_{j}\left(t_{k}\right)\right)-{\bar{k}}_{j}\left(z_{j}\left(t\right)\right)\right], \end{array}$$

so, we can get


(41) $$\begin{array}{l} d_{i}\eta_{i}\left(t\right)\leq \overset{n}{\underset{j=1}{\sum }}p_{ij}\left(t\right)H_{j}e_{j}\left(t\right)+\overset{n}{\underset{j=1}{\sum }}q_{ij}\left(t\right)K_{j}\eta_{j}\left(t\right), \end{array}$$

we can also get


(42) $$\begin{array}{l} d_{i}\left(t\right)|\eta_{i}\left(t\right)|\leq \left[p_{jj}^{+}\left(t\right)H_{j}+\underset{j\neq i}{\sum }|p_{ij}\left(t\right)|H_{j}\right]|e_{j}\left(t\right)|\\ \;+\left[q_{jj}^{+}\left(t\right)K_{j}+\underset{j\neq i}{\sum }|q_{ij}\left(t\right)|K_{j}\right]|\eta_{j}\left(t\right)|, \end{array}$$

so,


(43) $$\begin{array}{l} \left|\eta_{j}\left(t\right)|\leq \frac{p_{jj}^{+}\left(t\right)H_{j}+\underset{j\neq i}{\sum }|p_{ij}\left(t\right)|H_{j}}{d_{j}\left(t\right)-q_{jj}^{+}\left(t\right)K_{j}-\underset{j\neq i}{\sum }|q_{ij}\left(t\right)|K_{j}}|e_{j}\left(t\right)\right|\\ \;=\sigma_{j}\left(t\right)|e_{j}\left(t\right)|. \end{array}$$

From (12) and (13), we can get


(44) $$\begin{array}{l} \;\frac{d\|w(t){\|}_{1}}{dt}\\ \leq -\sum_{j=1}^{n}[c_{j}(t)-F_{j}a_{jj}^{+}(t)-F_{j}\sum_{j\neq i}\frac{\varsigma_{i}}{\varsigma_{j}}|a_{ij}(t)|]\varsigma_{j}|w_{j}(t)|\\ \;+\sum_{j=1}^{n}[c_{j}(t)+F_{j}a_{jj}^{+}(t)+F_{j}\sum_{j\neq i}\frac{\varsigma_{i}}{\varsigma_{j}}|a_{ij}(t)|]\varsigma_{j}|e_{j}(t)|\\ \;+\sum_{j=1}^{n}G_{j}b_{jj}^{+}(t)+G_{j}\sum_{j\neq i}\frac{\varsigma_{i}}{\varsigma_{j}}|b_{ij}(t)|]\sigma_{j}(t)\varsigma_{j}|w_{j}(t)|\\ \;+\sum_{j=1}^{n}G_{j}b_{jj}^{+}(t)+G_{j}\sum_{j\neq i}\frac{\varsigma_{i}}{\varsigma_{j}}|b_{ij}(t)|]\sigma_{j}(t)\varsigma_{j}|e_{j}(t)|, \end{array}$$

which implies


(45) $$\begin{array}{l} \frac{d||w\left(t\right)|{|}_{1}}{dt}\leq -\mu_{j}\left(t\right)\overset{n}{\underset{j=1}{\sum }}\varsigma_{j}|w_{j}\left(t\right)|+M_{1}\overset{n}{\underset{j=1}{\sum }}\varsigma_{j}|e_{j}\left(t\right)|\\ \;+\delta v_{j}\left(t\right)\varsigma_{j}|w_{j}\left(t\right)|+\delta v\left(t\right)\varsigma_{j}|e_{j}\left(t\right)|\\ \;\leq \left[-\mu_{j}\left(t\right)+\delta v\left(t\right)\right]||w\left(t\right)|{|}_{1}\\ \;+\left[M_{1}+\delta v\left(t\right)\right]\hbar \left(t\right)\\ \;\leq -\varepsilon_{3}||w\left(t\right)||+\left(M_{1}+\delta M_{2}\right)\hbar \left(t\right), \end{array}$$

for *M*_1_, *M*_2_ > 0, then we have


(46) $$\begin{array}{l} \;||w\left(t\right)||\\ \leq ||w\left(t_{0}\right)||e^{-\varepsilon_{2}\left(t-t_{0}\right)}+\left(M_{1}+\delta M_{2}\right)\int_{t_{0}}^{t}e^{-\varepsilon_{2}\left(t-s\right)}||\hbar \left(s\right)||ds\\ =e^{-\varepsilon_{2}\left(t-t_{0}\right)}\left[||w\left(t_{0}\right)||+\left(M_{1}+\delta M_{2}\right)\int_{t_{0}}^{t}e^{\varepsilon_{2}\left(s-t_{0}\right)}||\hbar \left(s\right)||ds\right], \end{array}$$

for *s* ∈ [*t*_0_, *t*], *t* ∈ [*t*_*k*_, *t*_*k*+1_).

Based on the *L*′ *Hospital* rule, we have


(47) $$\begin{array}{l} \underset{t\to +\infty }{\lim}||w\left(t\right)||=\underset{t\to +\infty }{\lim}\frac{M_{1}+\delta M_{2}}{e^{\varepsilon_{2}\left(t-t_{0}\right)}}\int_{t_{0}}^{t}e^{\varepsilon_{3}\left(s-t_{0}\right)}||\hbar \left(s\right)||ds,\\ \;=\underset{t\to +\infty }{\lim}\frac{M_{1}+\delta M_{2}}{\varepsilon_{3}}||\hbar \left(t\right)||,\\ \;=0. \end{array}$$

This means that the system (4) achieve outer-synchronization. The proof is completed.

In system (27), the state measurement error is defined as


$$e_{i}\left(t\right)=w_{i}\left(t_{k}^{i}\right)-w_{i}\left(t\right),$$

and the state measurement of static equation is as follows:


$$\eta_{i}\left(t\right)=z_{i}\left(t_{k}^{i}\right)-z_{i}\left(t\right),$$

where $t\in \left[t_{k}^{i},t_{k+1}^{i}\right),i=1,\ldots ,n$ and *k* = 0, 1, 2, …. The push-based decentralized updating rule is given as follows.

**Theorem 4** Let ȷ(*t*) be a positive decreasing continuous function on [0, +∞) with ϕ(0) > 0. Set $t_{k+1}^{i}$ as the triggering time point such that


(48) $$\begin{array}{l} t_{k+1}^{i}=\underset{ı\geq t_{k}^{i}}{\sup}\left\{ı:|e_{i}\left(t\right)|\leq {ȷ}_{i}\left(t\right),\forall t\in \left(t_{k}^{i},ı\right)\right\}, \end{array}$$

for *i* = 1, ⋯ , *n* and *k* = 0, 1, 2, …, there exist ς_*i*_, if μ_*j*_(ξ, *t*) ≥ ε_4_,


$$\underset{t\to +\infty }{\lim}{ȷ}_{i}\left(t\right)=0,$$

then the system (4) reaches out-synchronization.

**Proof**. Consider *w*_*i*_(*t*) for any neuron *i*(*i* = 1, …, *n*) and ς_*i*_ > 0(*i* = 1, …, *n*)


(49) $$\begin{array}{l} \;\frac{d\|w(t){\|}_{1}}{dt}\\ =\sum_{i=1}^{n}\varsigma_{i}sign(w_{i}(t))\frac{dw_{i}(t)}{dt}\\ =\sum_{i=1}^{n}\varsigma_{i}sign(w_{i}(t))[-c_{i}(t)w_{i}(t_{k}^{i})+\sum_{j=1}^{n}a_{ij}(t){\bar{f}}_{j}(w_{j}(t_{k}^{j}))\\ \;+\sum_{j=1}^{n}b_{ij}(t){\bar{g}}_{j}(z_{j}(t_{k}^{j}))],\\ =\sum_{i=1}^{n}\varsigma_{i}sign(w_{i}(t))[-c_{i}(t)w_{i}(t_{k}^{i})+\sum_{j=1}^{n}a_{ij}(t){\bar{f}}_{j}(w_{j}(t))\\ \;+\sum_{j=1}^{n}b_{ij}(t){\bar{g}}_{j}(z_{j}(t))]\\ \;+\sum_{i=1}^{n}\varsigma_{i}sign(w_{i}(t))[-c_{i}(w_{i}(t_{k}^{i})-w_{i}(t))]\\ \;+\sum_{i=1}^{n}\varsigma_{i}sign(w_{i}(t))\sum_{j=1}^{n}a_{ij}(t)[{\bar{f}}_{j}(w_{j}(t_{k}^{j}))-{\bar{f}}_{j}(w_{j}(t))]\\ \;+\sum_{i=1}^{n}\varsigma_{i}sign(w_{i}(t))\sum_{j=1}^{n}b_{ij}(t)[{\bar{g}}_{j}(z_{j}(t_{k}^{j}))-{\bar{g}}_{j}(z_{j}(t))], \end{array}$$

from (49), it holds


(50) $$\begin{array}{l} \;\frac{d||w\left(t\right)|{|}_{1}}{dt}\\ =\overset{n}{\underset{i=1}{\sum }}\varsigma_{i}sign\left(w_{i}\left(t\right)\right)\frac{d||w_{i}\left(t\right)||}{dt}\\ \leq -\overset{n}{\underset{i=1}{\sum }}\varsigma_{i}c_{i}|w_{i}\left(t\right)|+\varsigma_{i}c_{i}|e_{i}\left(t\right)|+\overset{n}{\underset{i=1}{\sum }}\varsigma_{i}a_{ii}^{+}\left(t\right)F_{i}|w_{i}\left(t\right)|\\ \;+\overset{n}{\underset{i=1}{\sum }}\varsigma_{i}b_{ii}^{+}\left(t\right)G_{i}|z_{i}\left(t\right)|+\overset{n}{\underset{i=1}{\sum }}\varsigma_{i}a_{ii}^{+}\left(t\right)F_{i}|e_{i}\left(t\right)|\\ \;+\overset{n}{\underset{i=1}{\sum }}\varsigma_{i}b_{ii}^{+}\left(t\right)G_{i}|\eta_{i}\left(t\right)|+\overset{n}{\underset{j=1}{\sum }}\underset{j\neq i}{\sum }\varsigma_{i}|a_{ij}|F_{j}|w_{j}\left(t\right)|\\ \;+\overset{n}{\underset{j=1}{\sum }}\underset{j\neq i}{\sum }\varsigma_{i}|a_{ij}|F_{j}|e_{j}\left(t\right)|+\overset{n}{\underset{j=1}{\sum }}\underset{j\neq i}{\sum }\varsigma_{i}|b_{ij}|G_{j}|z_{j}\left(t\right)|\\ \;+\overset{n}{\underset{j=1}{\sum }}\underset{j\neq i}{\sum }\varsigma_{i}|b_{ij}|G_{j}|\eta_{j}\left(t\right)|\\ \leq -\overset{n}{\underset{j=1}{\sum }}\left[c_{j}-F_{j}a_{jj}^{+}\left(t\right)-F_{j}\underset{j\neq i}{\sum }\frac{\varsigma_{i}}{\varsigma_{j}}|a_{ij}\left(t\right)|\right]\varsigma_{j}|w_{j}\left(t\right)|\\ \;+\overset{n}{\underset{j=1}{\sum }}\left[c_{j}+F_{j}a_{jj}^{+}\left(t\right)+\underset{j\neq i}{\sum }\frac{\varsigma_{i}}{\varsigma_{j}}|a_{ij}\left(t\right)|\right]\varsigma_{j}|e_{j}\left(t\right)|\\ \;+\left[G_{j}b_{jj}^{+}\left(t\right)+G_{j}\underset{j\neq i}{\sum }\frac{\varsigma_{i}}{\varsigma_{j}}|b_{ij}\left(t\right)|\right]\varsigma_{j}|z_{j}\left(t\right)|\\ \;+\left[G_{j}b_{jj}^{+}\left(t\right)+G_{j}\underset{j\neq i}{\sum }\frac{\varsigma_{i}}{\varsigma_{j}}|b_{ij}\left(t\right)|\right]\varsigma_{j}|\eta_{j}\left(t\right)|. \end{array}$$

For static equation, we have


(51) $$\begin{array}{l} \;d_{i}\left(z_{i}\left(t_{k}^{j}\right)-z_{i}\left(t\right)\right)\\ =\overset{n}{\underset{j=1}{\sum }}p_{ij}\left(t\right)\left[{\bar{h}}_{j}\left(w_{j}\left(t_{k}^{j}\right)\right)-{\bar{h}}_{j}\left(w_{j}\left(t\right)\right)\right]\\ \;+\overset{n}{\underset{j=1}{\sum }}q_{ij}\left(t\right)\left[{\bar{k}}_{j}\left(z_{j}\left(t_{k}^{j}\right)\right)-{\bar{k}}_{j}\left(z_{j}\left(t\right)\right)\right], \end{array}$$

so, we can get


(52) $$\begin{array}{l} d_{i}\eta_{i}\left(t\right)\leq \overset{n}{\underset{j=1}{\sum }}p_{ij}\left(t\right)H_{j}e_{j}\left(t\right)+\overset{n}{\underset{j=1}{\sum }}q_{ij}\left(t\right)K_{j}\eta_{j}\left(t\right), \end{array}$$

we also can get


(53) $$\begin{array}{l} d_{i}|\eta_{i}\left(t\right)|\leq \left[p_{jj}^{+}H_{j}+\underset{j\neq i}{\sum }|p_{ij}\left(t\right)|H_{j}\right]|e_{j}\left(t\right)|\\ \;+\left[q_{jj}^{+}K_{j}+\underset{j\neq i}{\sum }|q_{ij}\left(t\right)|K_{j}\right]|\eta_{j}\left(t\right)|, \end{array}$$

so,


(54) $$\begin{array}{l} \left|\eta_{j}\left(t\right)|\leq \frac{p_{jj}^{+}\left(t\right)H_{j}+\underset{j\neq i}{\sum }|p_{ij}\left(t\right)|H_{j}}{d_{j}-q_{jj}^{+}\left(t\right)K_{j}-\underset{j\neq i}{\sum }|q_{ij}\left(t\right)|K_{j}}|e_{j}\left(t\right)\right|\\ \;=\sigma_{j}\left(t\right)|e_{j}\left(t\right)|. \end{array}$$

From (50) and (54), we can get


(55) $$\begin{array}{l} \;\frac{d\|w(t){\|}_{1}}{dt}\\ \leq -\sum_{j=1}^{n}[c_{j}-F_{j}a_{jj}^{+}-F_{j}\sum_{j\neq i}\frac{\varsigma_{i}}{\varsigma_{j}}|a_{ij}(t)|]\varsigma_{j}|w_{j}(t)|\\ \;+\sum_{j=1}^{n}[c_{j}+F_{j}a_{jj}^{+}+F_{j}\sum_{j\neq i}\frac{\varsigma_{i}}{\varsigma_{j}}|a_{ij}(t)|]\varsigma_{j}|e_{j}(t)|\\ \;+\sum_{j=1}^{n}G_{j}b_{jj}^{+}+G_{j}\sum_{j\neq i}\frac{\varsigma_{i}}{\varsigma_{j}}|b_{ij}(t)|]\sigma_{j}(t)\varsigma_{j}|w_{j}(t)|\\ \;+\sum_{j=1}^{n}G_{j}b_{jj}^{+}+G_{j}\sum_{j\neq i}\frac{\varsigma_{i}}{\varsigma_{j}}|b_{ij}(t)|]\sigma_{j}(t)\varsigma_{j}|e_{j}(t)|, \end{array}$$

which implies


(56) $$\begin{array}{l} \frac{d||w\left(t\right)|{|}_{1}}{dt}\leq -\mu_{1}\left(t\right)\overset{n}{\underset{j=1}{\sum }}\varsigma_{j}|w_{j}\left(t\right)|+M_{1}\overset{n}{\underset{j=1}{\sum }}\varsigma_{j}|e_{j}\left(t\right)|\\ \;+\delta v\left(t\right)\varsigma_{j}|w_{j}\left(t\right)|+\delta v\left(t\right)\varsigma_{j}|e_{j}\left(t\right)|\\ \;\leq \left[-\mu_{j}\left(t\right)+\delta v\left(t\right)\right]||w\left(t\right)|{|}_{1}\\ \;+\left[M_{1}+\delta M_{2}\right]\overset{n}{\underset{j=1}{\sum }}\varsigma_{j}{ȷ}_{j}\left(t\right)\\ \;\leq -\varepsilon_{4}||w\left(t\right)||+\left[M_{1}+\delta M_{2}\right]||ȷ\left(t\right)||, \end{array}$$

then, we have


(57) $$\begin{array}{l} \left\|w(t)\|\leq \|w(t_{0}^{i})\|e^{-\varepsilon_{4}(t-t_{0}^{i})}+(M_{1}+\delta M_{2}\right)\\ \;\int_{t_{0}^{i}}^{t}e^{-\varepsilon_{4}(t-s)}\|(s)\|ds\\ \;=e^{-\varepsilon_{4}(t-t_{0}^{i})}[\|w(t_{0}^{i})\|+(M_{1}+\delta M_{2})\\ \;\int_{t_{0}^{i}}^{t}e^{\varepsilon_{4}(s-t_{0}^{i})}\|(s)\|ds], \end{array}$$

for $s\in \left[t_{0}^{i},t\right],t\in \left[t_{k}^{i},t_{k+1}^{i}\right)$.

Based on the *L*′*Hospital* rule, we have


(58) $$\begin{array}{l} \underset{t\to +\infty }{\lim}||w\left(t\right)||=\underset{t\to +\infty }{\lim}\frac{M_{1}+\delta M_{2}}{e^{\varepsilon_{4}\left(t-t_{0}^{i}\right)}}\int_{t_{0}}^{t}e^{\varepsilon_{4}\left(s-t_{0}^{i}\right)}||ȷ\left(s\right)||ds,\\ \;=\underset{t\to +\infty }{\lim}\frac{M_{1}+\delta M_{2}}{\varepsilon_{4}}||ȷ\left(t\right)||,\\ \;=0. \end{array}$$

The proof is completed.

**Remark 3.4** Theorems 3 and 4 make use of centralized and decentralized data sampling principles by relying on state variables. The systematic errors are used to estimate the character of system synchronization.

**Remark 3.5** Sampling control is a hot topic that cannot be ignored in the field of control theory. The significance of the control method lies in the size of the sampling interval and trigger condition. As long as the trigger conditions are met, information transmission can be started.

**Remark 3.6** In this paper, we study the outer-synchronization of ARTNNs, which are described by DAS. The DAS here is the system of index-1. The method is to build an acceptable sampling mechanism to make the system achieve outer-synchronization, where the sample interval is fixed and bounded. Therefore, in light of this work, the following next research directions are suggested: (1) Higher index DASs can be used as future research directions. (2) Intermittent sampling and random sampling can be used as sampling mechanisms. (3) Fractional systems can be considered.

**Remark 3.7** The research purpose of this paper is to create a workable sampling technique that will enable the system to achieve outer-synchronization. The approach does have certain drawbacks. (1) High index systems cannot use this strategy; it is only applicable to DASs with index-1. Differential equations cannot be created linearly from high index DASs. (2) Many of the equalities in the study require the upper and lower bounds, and the excitation function in the system must satisfy constraints that are equivalent to or even more stringent than the Lipschitz condition. (3) Integral inequalities are used to draw inferences utilizing data sampling techniques that heavily rely on the system's structure. As a result, the system's model structure imposes a major restriction on the approach used in this research.

## 4. A numerical example

In this section, a numerical simulation demonstrates the effectiveness of the conclusions.

### 4.1. Example description

**Example**.


(59) $$\{\begin{array}{c} \frac{dx_{1}(t)}{dt}=-c_{1}(t)x_{1}(t)+a_{11}(t)f_{1}(x_{1}(t))+a_{12}(t)f_{2}(x_{2}(t))\\ \;+b_{11}(t)g_{1}(y_{1}(t))+b_{12}(t)g_{2}(y_{2}(t))+J_{1}\\ \;0=-d_{1}(t)y_{1}(t_{)+p_{11}(}t)h_{1}(x_{1}(t))+p_{12}(t)h_{2}(x_{2}(t))\\ \;+q_{11}(t)k_{1}(y_{1}(t))+q_{12}(t)k_{2}(y_{2}(t))+I_{1}\\ \frac{dx_{2}(t)}{dt}=-c_{2}(t)x_{2}(t)+a_{21}(t)f_{1}(x_{1}(t))+a_{22}(t)f_{2}(x_{2}(t))\\ \;+b_{21}(t)g_{1}(y_{1}(t))+b_{22}(t)g_{2}(y_{2}(t))+J_{2}\\ \;0=-d_{2}(t)y_{2}(t)+p_{21}(t)h_{1}(x_{1}(t))+p_{22}(t)h_{2}(x_{2}(t))\\ \;+q_{21}(t)k_{1}(y_{1}(t))+q_{22}(t)k_{2}(y_{2}(t))+I_{2} \end{array}$$

$$\begin{array}{l} A=\left(\begin{array}{cc} a_{11}\left(t\right) & a_{12}\left(t\right)\\ a_{21}\left(t\right) & a_{22}\left(t\right) \end{array}\right)=\left(\begin{array}{cc} -1.2 & 0\\ 0 & 1.2 \end{array}\right),\\ B=\left(\begin{array}{cc} b_{11}\left(t\right) & b_{12}\left(t\right)\\ b_{21}\left(t\right) & b_{22}\left(t\right) \end{array}\right)=\left(\begin{array}{cc} 0.3 & -0.6\\ -1.2 & -0.2 \end{array}\right),\\ P=\left(\begin{array}{cc} p_{11}\left(t\right) & p_{12}\left(t\right)\\ p_{21}\left(t\right) & p_{22}\left(t\right) \end{array}\right)=\left(\begin{array}{cc} 1.4 & 0\\ 0 & 1.4 \end{array}\right),\\ Q=\left(\begin{array}{cc} q_{11}\left(t\right) & q_{12}\left(t\right)\\ q_{21}\left(t\right) & q_{22}\left(t\right) \end{array}\right)=\left(\begin{array}{cc} 0.3 & -0.6\\ -1.2 & -0.2 \end{array}\right),\\ C=\left(\begin{array}{c} c_{1}\left(t\right)\\ c_{2}\left(t\right) \end{array}\right)=\left(\begin{array}{c} 1.6\\ 1.6 \end{array}\right),\\ D=\left(\begin{array}{c} d_{1}\left(t\right)\\ d_{2}\left(t\right) \end{array}\right)=\left(\begin{array}{c} -0.5\\ -0.5 \end{array}\right),\\ J=\left(\begin{array}{c} J_{1}\\ J_{2} \end{array}\right)=\left(\begin{array}{c} 0.2\\ 0.2 \end{array}\right),\\ I=\left(\begin{array}{c} I_{1}\\ I_{2} \end{array}\right)=\left(\begin{array}{c} 0.1\\ 0.1 \end{array}\right),\\ f\left(x\right)=h\left(x\right)=\frac{1}{1+e^{-x}},g\left(y\right)=k\left(y\right)=\frac{1}{1+e^{-y}}, \end{array}$$

then, let $F_{j}=G_{j}=H_{j}=K_{j}=\frac{1}{2}$ and ξ_1_ = ξ_2_ = 1. We can calculate that,


$$\begin{array}{l} \max\sup\left\{\mu_{j}\left(\xi ,t\right)=c_{j}\left(t\right)-F_{j}\overset{+}{\underset{jj}{a}}\left(t\right)-F_{j}\frac{\varsigma_{i}}{\varsigma_{j}}|a_{ij}|\right\}=1.81\\ \min\left\{\delta_{j}\left(t\right)\right\}=\frac{p_{jj}^{+}\left(t\right)H_{j}+H_{j}\underset{i\neq j}{\sum }|p_{ij}\left(t\right)|}{d_{j}\left(t\right)-q_{jj}^{+}\left(t\right)-K_{j}\underset{i\neq j}{\sum }|q_{ij}\left(t\right)|}=-0.7,\\ \max\left\{v_{j}\left(\xi ,t\right)\right\}=G_{j}b_{jj}^{+}\left(t\right)+G_{j}\underset{i\neq j}{\sum }\frac{\varsigma_{i}}{\varsigma_{j}}|b_{ij}\left(t\right)|=0.75,\\ M_{1}=\underset{i\leq j\leq n}{\max}\underset{t\geq t_{0}}{\sup}\left\{c_{j}\left(t\right)+F_{j}a_{jj}^{+}\left(t\right)+F_{j}\underset{j\neq i}{\sum }\frac{\varsigma_{i}}{\varsigma_{j}}|a_{ij}\left(t\right)|\right\}=1.6,\\ M_{2}=\underset{i\leq j\leq n}{\max}\underset{t\geq t_{0}}{\sup}\left\{G_{j}b_{jj}^{+}\left(t\right)+G_{j}\underset{i\neq j}{\sum }\frac{\varsigma_{i}}{\varsigma_{j}}|b_{ij}\left(t\right)|\right\}=0.75, \end{array}$$

then,


$$\sup\left\{\mu_{j}\left(\xi ,t\right)-\delta v_{j}\left(\xi ,t\right)\right\}=1.81-\left(-0.5\right)*0.75=2.185,$$

Fix *N* = 2.2, ε_0_ = 0.5. Then, ε_*a*_ = 0.7, ε_*b*_ = 0.2, and the following inequality holds by calculation,


$$N\varepsilon_{a}\leq \varepsilon_{0}\left(2-\varepsilon_{a}\right),N\varepsilon_{b}\leq \varepsilon_{0}.$$

Fix $ȷ\left(t\right)=\hbar \left(t\right)=\frac{1}{t+1}$, satisfied


$$\begin{array}{l} \underset{t\to +\infty }{\lim}ȷ\left(t\right)=0,\underset{t\to +\infty }{\lim}\hbar \left(t\right)=0,\text{and}\underset{t\to +\infty }{\lim}\phi_{2}\left(t\right)=0. \end{array}$$

### 4.2. Simulation results

**Figure 1:**
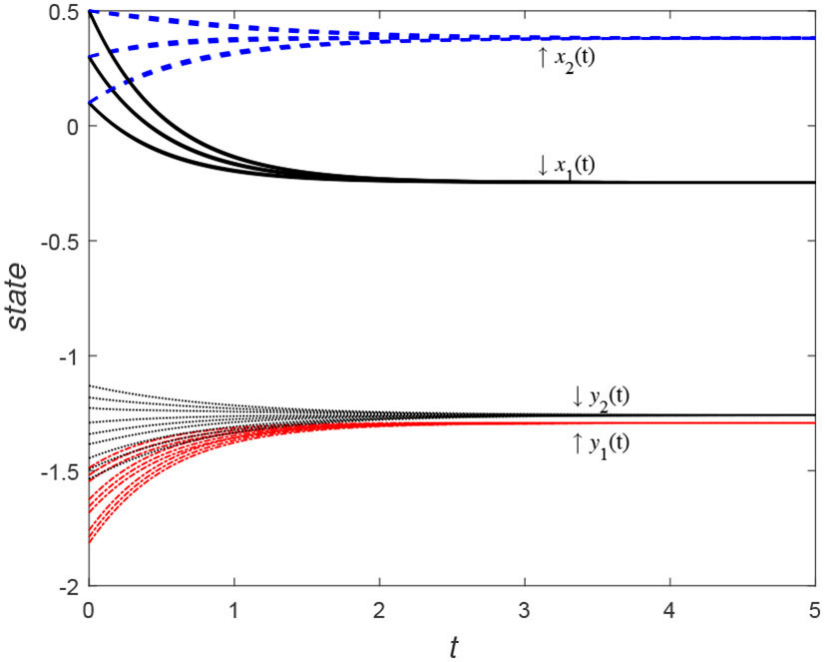
States *x*_1_(*t*) and *x*_2_(*t*) of model (59) with consistent initial values which *x*_1_(0), *x*_2_(0) ∈ {0.1, 0.3, and 0.5}.

In Figure 1, the state variable (*x*_1_(*t*), *x*_2_(*t*)) takes nine sets of initial values in turn and they are (0.1, 0.1), (0.1, 0.3), (0.1, 0.5), (0.3, 0.1), (0.3, 0.3), (0.3, 0.5), (0.5, 0.1), (0.5, 0.3), and (0.5, 0.5). Based on AEs of the model (59), the value of an algebraic variable (*y*_1_(*t*), *y*_2_(*t*)) is certain. It can be seen from the figure that the state function curve starting from any initial value point reaches the outer-synchronization.

**Figure 2:**
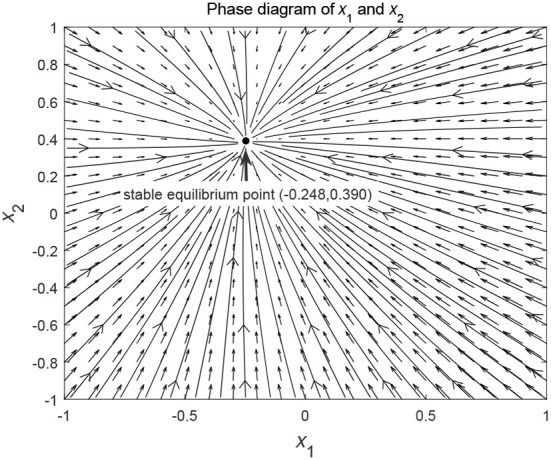
Phase diagram of differential states of model (59).

From a geometrical point of view, this means that the state function from any initial value will converge to the stable equilibrium point. When we consider a larger range of initial values, the same evolutionary trend can still be seen. Figure 2 shows that the phase curves (*x*_1_(*t*), *x*_2_(*t*)) from different initial values converge to the stable equilibrium point (−0.248, 0.390).

**Figure 3:**
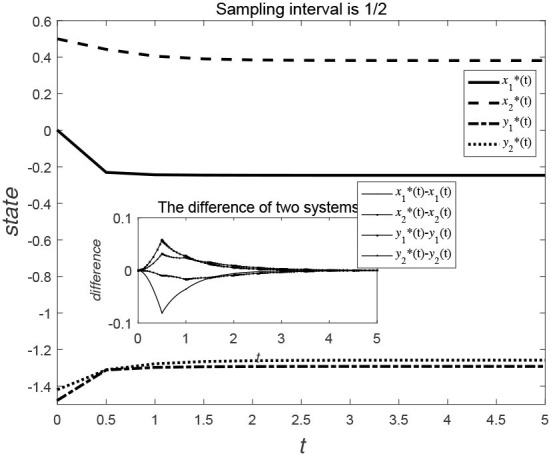
Evolution trend of state variables of the original system and the error system under centralized control style.

**Figure 4:**
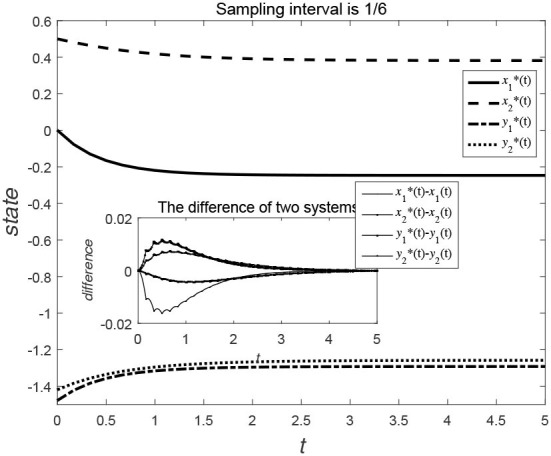
Evolution trend of state variables of original system and error system under centralized control style.

Consider the data-sampling principles, the data can be collected at certain time intervals for the time *t*. Figures 3, 4 show the evolution trend of the state function and system error when the sampling interval is $\frac{1}{2}$ and $\frac{1}{6}$. By comparing Figures 3, 4, it can be seen that when the sampling time interval is smaller, the error between the sampling system and the original system will be smaller, but outer-synchronization will be ultimately achieved.

**Figure 5:**
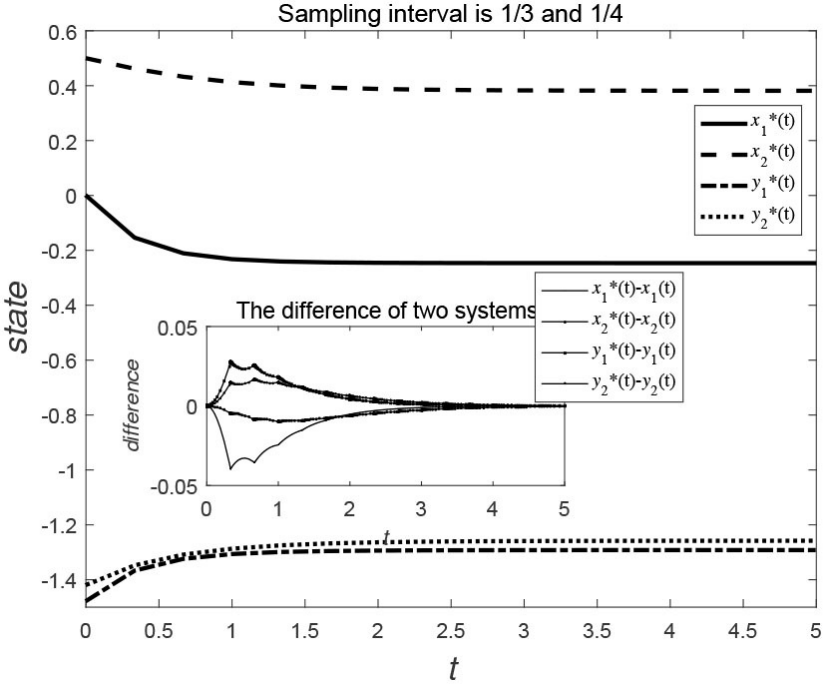
Evolution trend of state variables of original system and error system under decentralized control style.

**Figure 6:**
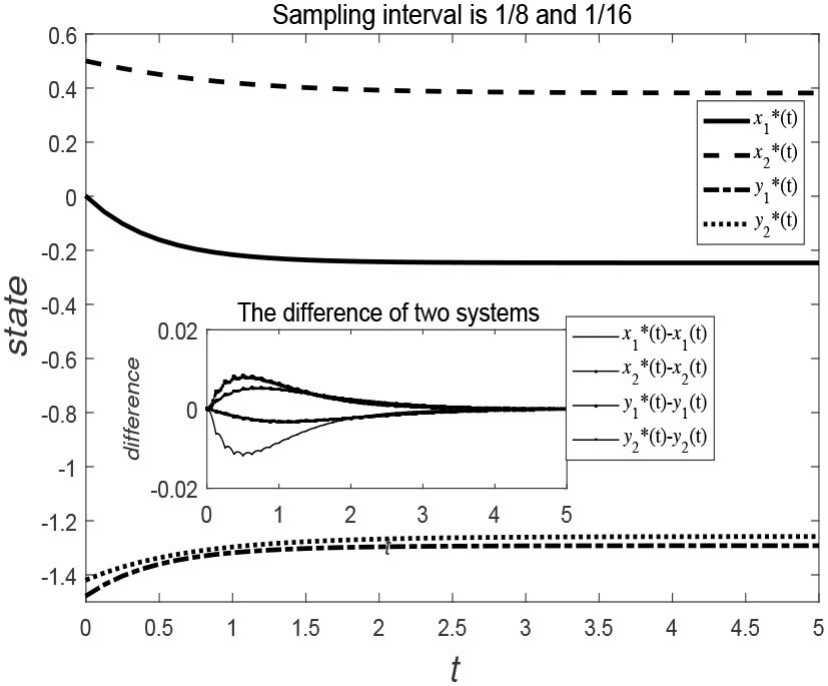
Evolution trend of state variables of the original system and the error system under centralized control style.

**Figure 7:**
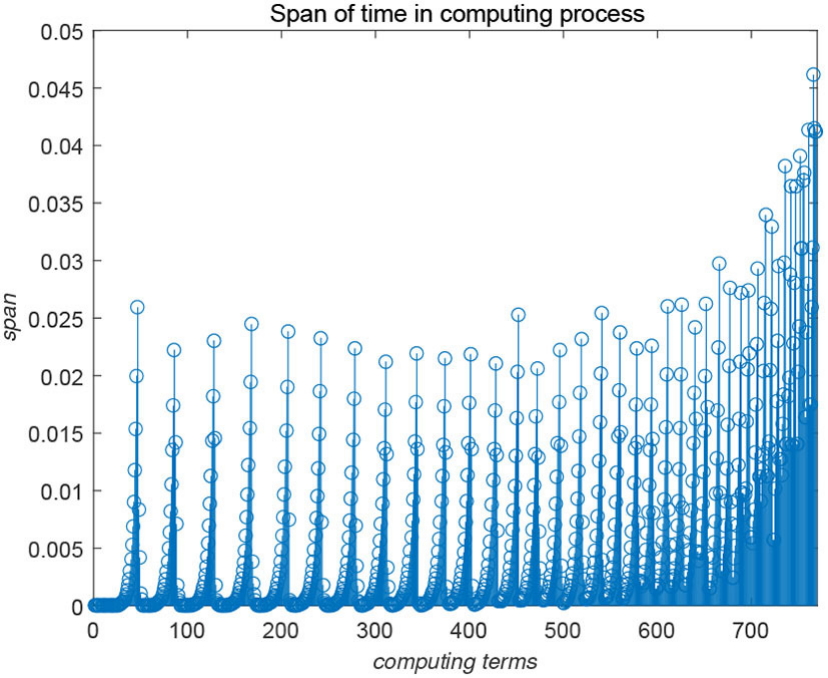
Release time point and release time interval.

Figures 5, 6 show the evolution trend of the state curve and the error range before and after systematic sampling under the decentralized data sampling principle, respectively. The sampling intervals of time *t* in Figure 5 are $\frac{1}{3}$ and $\frac{1}{4}$ and those in Figure 6 are $\frac{1}{8}$ and $\frac{1}{16}$. Comparing Figures 5, 6, it is also observed that when the sampling time interval is smaller, the error between the sampling system and the original system will be smaller, but outer-synchronization will be achieved in the end. Figure 7 shows the release time point and release time interval.

It can be observed from the simulation results that no matter which sampling method is used, as the conditions of Theorems 1 − 4 are satisfied, the system can be reached outer-synchronized with the premise of more cost savings.

### 4.3. Simulation steps

The numerical simulation in this section is carried out according to the following steps:

**Step 1** Define the original NN described by DAS, where the independent variable of the state function is a continuous-time variable *t*.
**Step 2** Determine the initial values of state variables and their derivatives and check the initial value compatibility.
**Step 3** The ARTNN model represented by the DAS is regarded as an implicit DE system, and the solutions of the original system are solved by using the implicit DE.
**Step 4** Define the sampling function and replace the variables *t* in the original system.
**Step 5** By derivation, the AEs in the original system (1) are transformed into equivalent DEs, and the original DAS (1) is transformed into the equivalent differential system (2).
**Step 6** Solving the equivalent differential System (2) by using a method of neutral-type time-delay DE.
**Step 7** Compare the solutions of the original DAS (1) and the equivalent differential system (2).

**Remark 4.1** The initial values of DAS (1) and the equivalent differential system (2) are the same, so the initial value of the solution in the sixth step is the same as the initial value of the original system in the second step.

## 5. Conclusion

In this research, we demonstrated that outer-synchronization of ARTNN may be achieved through the application of suitable centralized and decentralized data sampling procedures. These theoretical results enhanced and enriched relevant research already in existence. By establishing suitable sampling techniques, sufficient conditions for the outer-synchronization of the system are obtained in this study. The positive lower bound of the sampling interval ensured that the system will not encounter the Zeno phenomenon during the sampling procedure. This paper contains ideas for future discussion: (1) outer-synchronization of ARTNN taking both conservatism and complexity into account; (2) analysis of outer-synchronization of ARTNN subject to stochastic disturbance; (3) how to increase the sampling interval so that the results obtained by the error system are consistent with the original system.

## Data availability statement

The original contributions presented in the study are included in the article/supplementary material, further inquiries can be directed to the corresponding author.

## Author contributions

PL: numerical simulation and description. QL: drafting the manuscript. ZL: full text proofreading. All authors contributed to the article and approved the submitted version.

## Funding

This work was supported by the Natural Science Foundation of China under Grant 61773152 and Hubei Provincial Department of Education under Grant No. 2018CFB532.

## Conflict of interest

The authors declare that the research was conducted in the absence of any commercial or financial relationships that could be construed as a potential conflict of interest.

## Publisher's note

All claims expressed in this article are solely those of the authors and do not necessarily represent those of their affiliated organizations, or those of the publisher, the editors and the reviewers. Any product that may be evaluated in this article, or claim that may be made by its manufacturer, is not guaranteed or endorsed by the publisher.
